# Full-Arch Rehabilitation Using Trans-Mucosal Tissue-Level Implants with and without Implant-Abutment Units: A Case Report

**DOI:** 10.3390/dj10070116

**Published:** 2022-07-01

**Authors:** Massimo Carossa, Mario Alovisi, Armando Crupi, Giulia Ambrogio, Francesco Pera

**Affiliations:** Department of Surgical Sciences, CIR Dental School, University of Turin, Via Nizza 230, 10126 Turin, Italy; mario.alovisi@unito.it (M.A.); armando.crupi@unito.it (A.C.); giulia.ambrogio@unito.it (G.A.); francesco.pera@unito.it (F.P.)

**Keywords:** dental implants, tissue-level, convergent collar, full-arch rehabilitation, immediate loading, abutments, case report

## Abstract

Recently, tissue-level implants with a convergent collar have been introduced. While different studies have investigated the outcomes of this implant design in the rehabilitation of single teeth, its use in full-arch rehabilitation has yet to be investigated. The present case report describes the clinical outcomes of a full-arch immediate loading rehabilitation using tissue-level implants, with and without using implant-abutment units, with 2 years of follow-up. A female patient with mandibular terminal dentition and a high level of bone resorption (distal areas with a few millimeters of residual bone in the vertical dimension and both distal and anterior areas with narrow crestal bone in the horizontal dimension) was seen at the C.I.R Dental School, Turin, Italy. The patient was seeking to be rehabilitated with fixed prosthodontics, and she was found eligible for an immediate loading implant full-arch rehabilitation. Four implants were inserted in the same appointment. The two anterior implants were inserted straight and connected directly to the prosthesis (no abutments); the two distal implants were tilted in order to avoid the alveolar nerve and connected to two 30° angulated abutments. Two years post-implant placement, all of the implants were successfully integrated, resulting in an implant survival rate of 100%. The peri-implant soft tissues were stable at all the implant sites. No differences were highlighted between those implants with and without abutments. Within the limitations of the present clinical report, implant full-arch rehabilitations with tissue-level implants both with and without implant-abutment units showed optimal outcomes after two years of follow-up. Further research is encouraged to confirm whether this implant design may be a valid alternative to traditional implants in this type of rehabilitation, with or without implant-abutment units.

## 1. Introduction

Immediate loading full-arch rehabilitation has become a predictable rehabilitation, which is able to provide long-term success [1]. It allows for restoration within 24–48 h [2] in both the aesthetics and function of patients who are fully edentulous or with terminal residual dentition, producing a high level of satisfaction [3].

However, although immediate loading rehabilitation in fully edentulous patients is described as a predictable treatment [4,5], it is not without complications [6], and research is continuously focusing on how to improve its potential.

An implant abutment is a prosthetic component that is commonly screwed into the implant to connect it to the prosthesis. By bearing the occlusal forces, it protects the implant and the peri-implant tissues from excessive loads [7,8]. Moreover, the passive fit of the prosthesis is increased, both in single, multiple, and full-arch rehabilitation [9,10], and the prosthetic platform is moved far from the implant neck. This results in a reduced risk of bacteria contamination at the implant site and avoids damage to the soft tissue during the prosthetic phases [11]. Nevertheless, the introduction of angulated abutments introduced the possibility of tilting the distal implants in order to avoid the anatomic boundaries (alveolar nerve and maxillary sinus) [4]. In a prospective randomized clinical study, Göthberg et al. followed the outcomes of three unit implants placed either with or without abutments at 1 [12], 3 [10], and 5 [13] years of follow-up. Their results highlighted how the implants that were directly connected to the prosthesis (no abutments) were prone to higher bone resorption compared to the control groups.

However, despite all of the advantages described above, some authors have started to point out how the implant–abutment connection [14] and the implant collar morphology [15] may be considered critical factors for the prognosis of implant rehabilitation. In a recent systematic review, Tallarico et al. [16] highlighted that the area where the abutment is screwed into the implant may be prone to bacterial contamination due to the micro-gap between the implant head and the prosthetic components. In fact, two interfaces are created when an abutment is adopted: one between the implant and the abutment and one between the abutment and the prosthesis. These two interfaces may be prone to micro-gaps between the different components and, consequently, may be prone to bacteria colonization. This can play an important role in the development of inflammation of peri-implant tissues. For this reason, research in recent years has focused on the investigation of different implant–abutment connections [17,18], as well as different materials and topography for the transmucosal part of the abutment [19,20]. In a recent systematic review, Ceruso et al. [21] highlighted how the ideal implant–abutment connection should be considered as a one-piece implant where only one interface is created with the prosthesis.

Recently, tissue-level implants with a convergent collar have been introduced in contrast with traditional tissue-level implants that have divergent collars. According to Panadero et al. [22], the convergent collar offers better and more predictable results in terms of aesthetic outcomes and soft tissue adaptation. Moreover, it shows less peri-implant bone loss compared to the divergent design. To date, different studies [23,24] investigated the outcome of tissue-level implants with a convergent collar in the short/medium term, showing positive findings. However, the majority of the studies report their use in the rehabilitation of single teeth, mainly in the aesthetic area. To the authors’ knowledge, no previous studies have investigated the clinical outcomes of tissue-level implants with convergent collars in full-arch rehabilitations.

The aims of the present case report are: (1) to describe the clinical outcomes of a full-arch implant rehabilitation using transmucosal tissue-level implants with and without implant–abutment units; (2) to discuss whether this new implant design may be used without implant-abutment units, as an alternative to the traditional implant–abutment-prosthesis procedure.

## 2. Case Report

The present case report was described following the CARE Checklist (https://www.care-statement.org/checklist, accessed on 10 June 2022).

**Figure 1:**
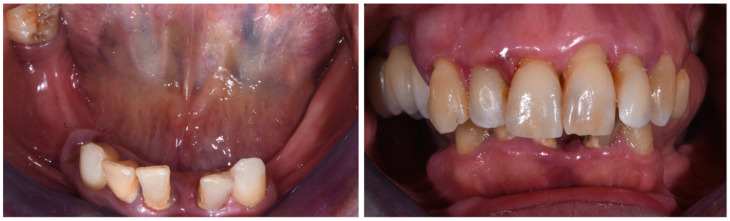
Mandibular assessment. Left: Occlusal view. Right: Frontal view (January 2020).

**Figure 2:**
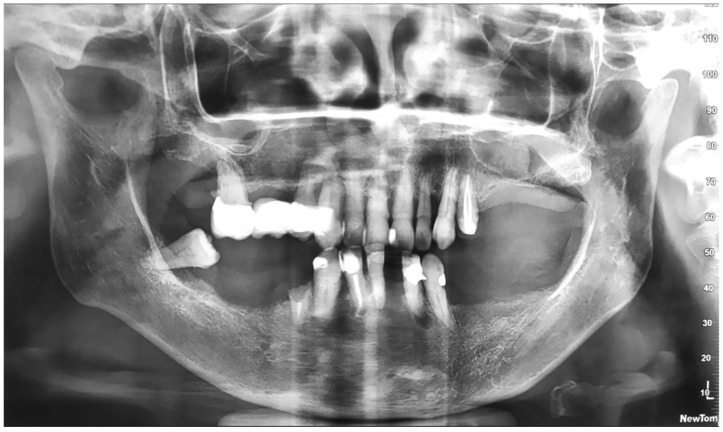
Panoramic radiograph (January 2020).

A female patient, 49 years old, non-smoker, and healthy, was referred to the Prosthodontic and Implant Department, C.I.R Dental School, University of Turin, in January 2020 for the evaluation and rehabilitation of the lower jaw (Figure 1 and Figure 2).

The patient was wearing a lower partial removable prosthesis. Her chief complaint was the following: (1) to be rehabilitated, if possible, with fixed prostheses; (2) not to remain for a long time without the teeth.

**Figure 3:**
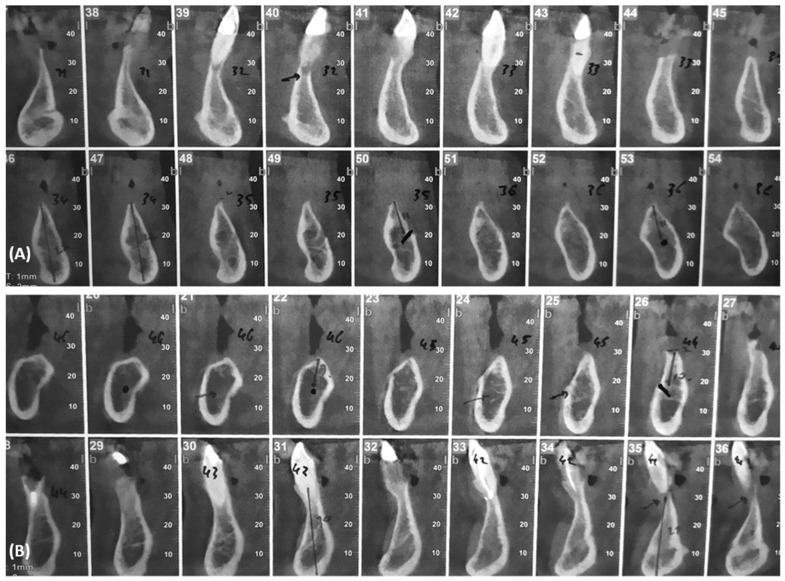
Tc cone bean. (**A**) Left side. (**B**) Right side (February 2020).

After the examination of the TC cone Bean (Figure 3), the lower remaining teeth were considered to have an unfavorable prognosis.

The treatment plan included: extraction of the hopeless teeth, implant insertion, and post-extractive implant insertion using two upright implants in the anterior area and two tilted implants in the posterior area; and immediate loading with provisional screw-retained full-arch prosthesis.

### Surgery Appointment

The surgical and prosthetic protocols applied (Columbus Bridge Protocol) are described in detail in previously published papers [25].

Written informed consent was obtained from the patient.

The patient underwent scaling and root planning two days prior to the surgery in order to lower the bacterial load of the mouth and predispose the best conditions for surgery and optimal healing of the tissues.

**Figure 4:**
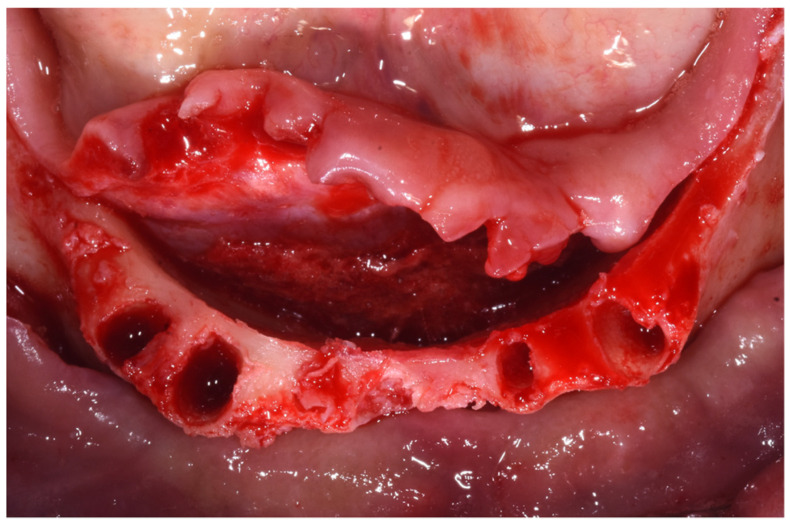
Mucoperiosteal flap elevation after extraction of the hopeless teeth. (March 2020).

Preoperative antibiotic coverage with Amoxicillin 875 mg + Clavulanic acid 125 mg every 12 h for 6 days was prescribed starting 1 day prior to the surgery. The surgery was performed under local anesthesia (4% articaine with 1:10,000 adrenaline; Alfacaina SP; Dentsply Italy, Rome, Italy). All of the remaining hopeless teeth were extracted on the day of surgery, and a full-thickness mucoperiosteal flap was elevated (Figure 4).

**Figure 5:**
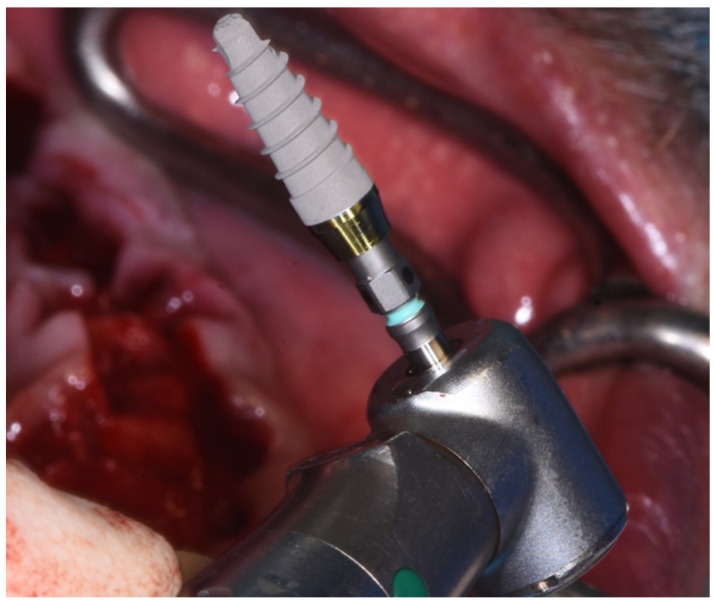
Prama RF implant.

Four titanium implants, 3.8 × 15 mm in length and with the same macro- and micro-topography (Prama RF, Sweden & Martina, Due Carrare, Padova, Italy) (Figure 5) were inserted.

**Figure 6:**
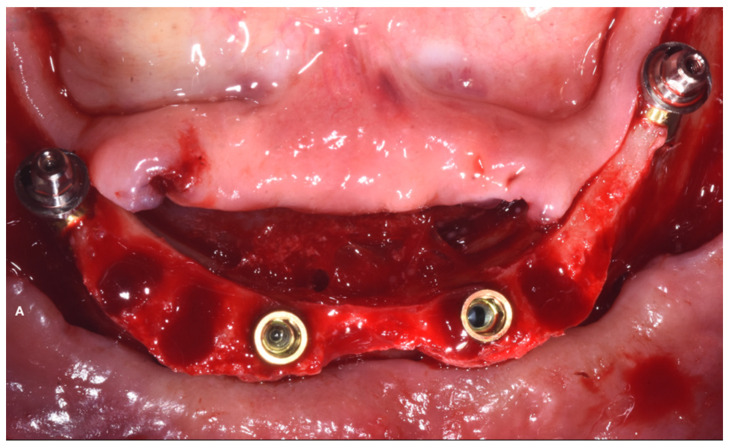
Implant insertion. The two distal implants are connected to two 30° angulated abutments, while the two anterior implants are with no abutments.

The two anterior implants were inserted straight, while the two posterior ones were tilted in order to avoid the anatomical boundary represented by the alveolar nerve. Two 30° inclined abutment units (Abutment P.A.D 330-303, Sweden & Martina, Due Carrare, Padova, Italy) were screwed into the two distal implants, while the two anterior implants were left without implant–abutment units (Figure 6).

This implant is characterized by an internal hexagon connection with a 3.4-mm diameter and a conically shaped titanium collar, 2.8-mm high, tapered in the occlusal direction. The collar is machined with the UTM (Ultrathin Threaded Microsurface) technique. The conical design of the implant body was preferred to the cylindrical one in order to maximize implant stability, allowing immediate loading.

The implant body presents the proprietary ZirTi surface (Zirconium sand-blasted acid-etched titanium).

**Figure 7:**
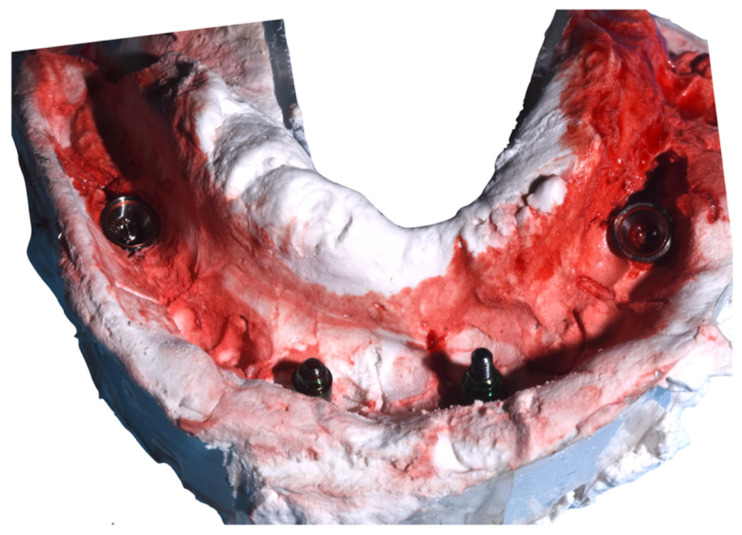
Plaster impression.

The sites were prepared with dedicated drills using Sweden & Martina surgical kit, and all the implants were inserted with a Torque insertion > 50 Ncm. Due to the good bone quality, no under-preparation was adopted. The implants were inserted with the treated portion placed 1 mm below the bone crest. Then, four transfers were screwed prior to suturing in order to allow the surgeon to check for the perfect fit with the implants. Two transfers were screwed directly on the anterior implants (L-TRA-380, Sweden & Martina, Due Carrare, Padova, Italy), and two transfers (PAD-TRA, Sweden & Martina, Due Carrare, Padova, Italy) were screwed on the abutments for the posterior tilted implants. Eight interrupted sutures were made using non-absorbable multi-filament sutures (PERMA-HAND SILK 3-0; Ethicon, Somerville, NJ, USA). All of the transfers were impressed with the open tray technique using impression plaster (BF-plaster Dental, Torino, Italy) (Figure 7).

Two trans-mucosal healing cups (Trans-mucosal healing cups Prama IN; Sweden & Martina, Due Carrare, Padova, Italy) were then screwed to cover the two anterior implants, and two abutment covers (PAD-CG, Sweden & Martina, Due Carrare, Padova, Italy) were then screwed on the two distal implants. The patient was then given postoperative instructions, including a 0.2% chlorhexidine digluconate (Corsodyl, GlaxoSmithKline, Verona, Italy) mouth rinse twice a day for two weeks starting the day after surgery, pain killers when needed, diet, and hygienic recommendations.

**Figure 8:**
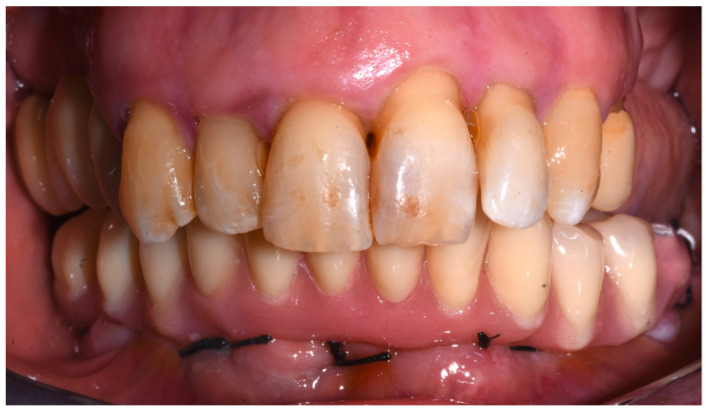
Provisional full-arch prosthesis.

After 48 h, a provisional full-arch prosthesis of 12 masticatory units was screwed (Figure 8), and the implants were loaded. The provisional prosthesis was made of resin with a metal framework, and the following occlusal scheme was adopted: (1) no cantilever; (2) pit-cusp tooth morphology reproducing the natural dentition; (3) point of contacts as homogeneous as possible, avoiding pre-contacts, interferences, and excessive load on single points.

**Figure 9:**
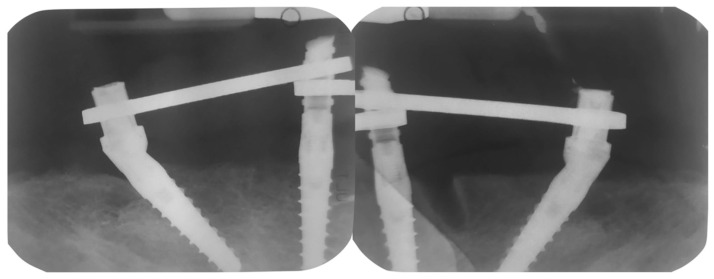
Implant X-rays immediately after load.

Periapical X-rays were acquired (T0) to check the correct positions of the implants and the provisional prosthesis (Figure 9).

**Figure 10:**
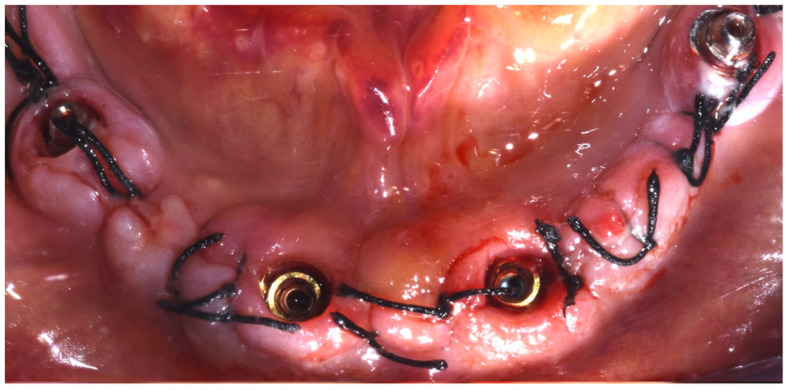
Soft tissue healing after 48 h.

The soft-tissue healing at 48 h after surgery is shown in Figure 10.

Sutures were removed one week after surgery, and recalls were planned at 2 weeks and 3, 6, 12, and 24 months. The occlusal scheme was carefully checked at each appointment.

Six months after the provisional load, the patient returned to realize the final prosthesis. A new open-tray impression using impression plaster (BF-plaster Dental, Torino, Italy) was taken, and the final prosthesis was fabricated. The final prosthesis was made in composite resin with a metal framework with the same occlusal scheme described for the provisional one.

**Figure 11:**
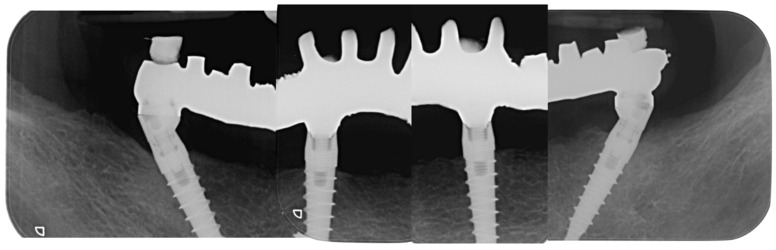
Implant X-rays immediately after load. (March 2022).

Intraoral X-rays were taken at the last check-up 24 months after surgery (Figure 11), and bone resorption and peri-implant soft tissue indexes (probing depth, bleeding on probing, and plaque) were recorded at 24 months (T24).

**Figure 12:**
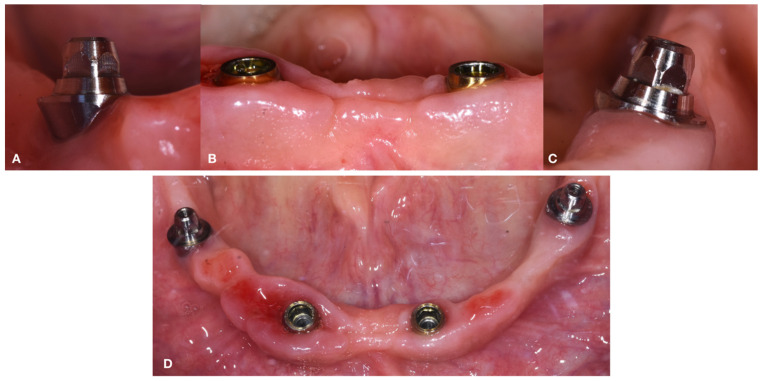
Details of soft tissue healed after 24 months. (March 2022) (**A**). Distal right implant. (**B**) Anterior implants. (**C**) Distal left implant. (**D**) Occlusal view.

Details of soft-tissue healing at 24 months are shown in Figure 12.

Bone reabsorption was measured using intraoral X-rays according to the method previously published by the authors [17]. The implant neck was taken as a reference point. The peri-implant bone level was measured as the distance between the reference points and the coronal bone on both the mesial and distal aspects of each implant. It was measured on the intraoral X-rays acquired 48 h after surgery (T0) and on the ones acquired two years after surgery (T24). The difference between T0 and T24 produced the bone level change.

Nonetheless, for long-term maintenance, the patient was then inserted into peri-implant oral hygiene recalls based on postbiotic gels and ozonized water, following the protocol previously described in published articles [26,27,28].

## 3. Results

No implant failed resulting, in an implant survival rate of 100% after 24 months of follow-up. The mean value for bone-level loss was 1.19 mm at 2 years post implant placement. The mean value for bone-level loss for the distal implants (with abutments) was 1.18 mm (mean of 1.2 mm on the mesial side and 1.2 mm on the distal one). The mean value for bone-level loss for the anterior implants (without abutments) was 1.2 mm (mean of 1.15 mm on the mesial side and 1.25 mm on the distal one).

The patient anecdotally reported being very satisfied with his implant rehabilitation.

The peri-implant soft tissue indexes at 24 months were the following:

- Probing depth (mm): Distal right (DR) implants showed 2 mm probing depth on the mesial side, 2 mm on the vestibular side, 3 mm on the distal side, and 3 mm on the lingual side. The anterior right (AR) implant showed 3 mm probing depth on the mesial side, 3 mm on the vestibular side, 2 mm on the distal side, and 3 mm on the lingual side. The anterior left (AL) implant showed 2 mm probing depth on the mesial side, 2 mm on the vestibular side, 3 mm on the distal side, and 3 mm on the lingual side. The distal left (DL) implant showed 2 mm probing depth on the mesial side, 2 mm on the vestibular side, 3 mm on the distal side, and 3 mm on the lingual side.
- Bleeding on probing (BOP): DR, AR, and DL implants presented one side each with BOP. No BOP was detected on the AL implant.
- No plaque was detected at any sites.

## 4. Discussion

The first aim of the present case report was to describe the clinical outcomes of full-arch implant rehabilitations using transmucosal tissue-level implants with convergent collars with and without implant–abutment units. The patient presented with a high level of bone resorption (distal areas with a few millimeters of residual bone in the vertical dimension and both distal and anterior areas with narrow crestal bone in the horizontal dimension) evaluated by the Tc Cone Bean. In order to avoid the anatomical boundaries represented by the alveolar nerve, the two distal implants were tilted, while the two anterior ones were inserted straight. Later, the two distal implants were connected to the angulated abutment, and the two anterior implants were connected directly to the prosthesis. After 24 months of follow-up, all the implants were successfully integrated, resulting in an implant survival rate of 100%. Healthy and stable soft tissues were observed around all the implants, and no prosthetic complications were observed. The rehabilitation was consequently considered successful. The second aim of the present case report was to discuss whether this new implant design may be used without implant–abutment units as an alternative to the traditional implant–abutment-prosthesis procedure. Simplifying the prosthetic procedure by not using the abutments may result in decreased costs for patients, prevention of micro-gasps due to the double interfaces, and facilitating the emergence profile of the prosthesis. However, the procedure must be supported by scientific results. No differences in terms of peri-implant soft and hard tissues were highlighted within the implants inserted in the same patient. Following the results obtained from the present case report, tissue-level implants seem to be a valid alternative to traditional implants in full-arch rehabilitations, both with and without abutments. However, no conclusions can be obtained. The results of comparing the two implants with abutment with the two implants without abutment in the same patients are limited to the single case presented. This is a limitation inherent to the design of the case report in the present article. A single case is not enough to provide scientific recognition for a specific technique since different variables may be presented when more cases are evaluated (e.g., parafunctional activities, medical conditions, different materials in the antagonist arches, etc.). In addition, the follow-up was limited, and the implants were inserted in two different areas (posterior vs. anterior). Consequently, the present results should be read with caution. However, case reports are useful for describing new techniques. The optimal results obtained from the present case report concerning the use of transmucosal tissue levels in full-arch rehabilitations and using them without abutment encourage further research on the topic. Further prospective cohort studies are required in order to confirm the outcomes.

To date, the only data available on this implant design are from single-implant rehabilitations, which are difficult to compare due to the numerous differences regarding several aspects of the rehabilitation, including the surgical and prosthodontic protocols applied and the parameters recorded. In a one-year clinical study, Castillo et al. [29] concluded that implants with transmucosal convergent collars were prone to less bone resorption when compared to implants with a cylindrical transmucosal neck. Canullo et al. [30] compared tissue level implants to traditional bone level implants in the rehabilitation of a single missed tooth in the aesthetic area up to 5 years of follow-up. The authors found statistically less bone resorption in favor of tissue-level implants.

Following the manufacturer’s instructions, the collar of this particular implant design can be inserted at both the bone- and tissue level. The deepness of implant insertion should be determined individually based on the clinical conditions evaluated by the clinicians. As described by Canullo et al. [24], the main advantages of the tissue-level implants with a convergent collar are: (1) the ability to seal the implant-abutment junction, avoiding a micro-gap at the trans-mucosal level, and (2) the increase in peri-implant soft tissue thickness by improving the space for the supra-crestal tissue. In addition, it has been hypothesized that the myofibroblasts’ contractile ability that occurs during second intention soft tissue healing around implants is favored by this specific implant design [31]. As a consequence, the use of a convergent implant should improve the thickness and stability of the soft tissue around the implant [32]. Having a stable and thick, soft tissue sealed around the implant is considered a fundamental factor for the long-term success and aesthetic outcome of the rehabilitation [33,34]. Other advantages of this new implant may be linked to the prosthesis platform. A prosthesis platform that is distant from the bone level may reduce both the bacterial contamination of the implant and the peri-implant mucosa trauma that possibly occurs during the unscrewing and screwing procedures. Internal hexagon connections have been reported to reduce the risk of bacterial contamination compared to external hexagon connections. However, Menini et al. [17], in a split-mouth study, found no statistical differences in peri-implant soft and hard tissue behavior between internal and external hexagon connections in full-arch implant rehabilitation.

Platform switching is another similar concept described to preserve the marginal bone level by increasing the thickness of the soft tissue [35]. By using an internal connection, it is possible to decrease the abutment diameter compared to the diameter of the implant head, thus improving the space available for peri-implant soft tissue. However, despite the different treatment options and advances [36,37] in the treatment of full-arch rehabilitations, research on the topic is still a pressing matter.

In conclusion, the data presented from the literature about this implant design are promising. The present article adds clinical reports about their use in full-arch rehabilitation, encouraging further research on the topic. Research on the abutment components is still open. Future cohort studies are needed to validate whether tissue-level implants without the use of the abutment may represent a valid alternative to the traditional workflow. If confirmed in further research, advancements on this topic may provide an alternative implant design for this type of rehabilitation, as well as simplify the prosthetic procedures and avoid multiple micro gaps by screwing the prosthesis directly into the implant collar.

## 5. Conclusions

Within the limitations of the present case report, the implant full-arch rehabilitation described in the present article with tissue-level implants, both with and without abutments, showed optimal outcomes after two years of follow-up. Further research is needed to confirm whether this implant design may be a valid alternative to traditional implants in this type of rehabilitation, with or without implant-abutment units.

## Data Availability

The datasets generated during and/or analysed during the current study are available from the corresponding author on reasonable request.
